# The Multiple Roles of Mps1 in *Drosophila* Female Meiosis

**DOI:** 10.1371/journal.pgen.0030113

**Published:** 2007-07-13

**Authors:** William D Gilliland, Stacie E Hughes, Jeffrey L Cotitta, Satomi Takeo, Youbin Xiang, R. Scott Hawley

**Affiliations:** 1 Stowers Institute for Medical Research, Kansas City, Missouri, United States of America; 2 Department of Physiology, University of Kansas Medical Center, Kansas City, Kansas, United States of America; Stanford University School of Medicine, United States of America

## Abstract

The *Drosophila* gene *ald* encodes the fly ortholog of *mps1,* a conserved kinetochore-associated protein kinase required for the meiotic and mitotic spindle assembly checkpoints. Using live imaging, we demonstrate that oocytes lacking Ald/Mps1 (hereafter referred to as Ald) protein enter anaphase I immediately upon completing spindle formation, in a fashion that does not allow sufficient time for nonexchange homologs to complete their normal partitioning to opposite half spindles. This observation can explain the heightened sensitivity of nonexchange chromosomes to the meiotic effects of hypomorphic *ald* alleles. In one of the first studies of the female meiotic kinetochore, we show that Ald localizes to the outer edge of meiotic kinetochores after germinal vesicle breakdown, where it is often observed to be extended well away from the chromosomes. Ald also localizes to numerous filaments throughout the oocyte. These filaments, which are not observed in mitotic cells, also contain the outer kinetochore protein kinase Polo, but not the inner kinetochore proteins Incenp or Aurora-B. These filaments polymerize during early germinal vesicle breakdown, perhaps as a means of storing excess outer kinetochore kinases during early embryonic development.

## Author Summary

Female meiosis is the process that ensures developing eggs (called oocytes*)* receive the proper complement of chromosomes. The failure to accurately segregate chromosomes results in aneuploidy, which is the leading cause of birth defects in humans. Cells contain checkpoints that help ensure proper chromosome segregation. Here, we present a study of the *Drosophila* homolog of *monopolar spindles 1 (mps1),* which is a key checkpoint component. Mutants in *mps1* produce oocytes with the wrong number of chromosomes. Using live imaging of female meiosis, we find that *mps1* mutants do not delay the cell cycle as is normally observed in wild-type flies. This delay gives chromosomes the time needed to properly align before cell division, and therefore the defect caused by *mps1* mutants is due to chromosomes being forced to segregate before they have had time to properly align. Additionally, we find that *mps1* as well as two other checkpoint proteins localize to numerous filaments throughout the oocyte. These filaments appear to form when the nuclear envelope breaks down, and disappear late in meiosis. While the function of these structures is not known, they appear similar to filaments seen in female meiosis in nematodes, and may be required to regulate these proteins.

## Introduction

Female meiosis in *Drosophila* has proved to be a useful model system for studying the mechanisms of chromosome segregation and the regulation of the meiotic cell cycle. While the canonical mitotic cell division entails the separation of sister chromatids, at the first meiotic division homologous centromeres segregate to opposite poles, followed by a more mitosis-like segregation of sister chromatids at meiosis II [1]. To ensure the production of euploid gametes, pairs of homologous chromosomes must therefore successfully perform three tasks: homologous chromosomes must be paired, that pairing must be maintained to hold chromosomes together, and finally the pairing must be dissolved to move homologous centromeres to opposite poles. While the formation of chiasmata through homologous recombination is normally necessary and sufficient to maintain pairing, *Drosophila* can accurately segregate achiasmate chromosomes through the distributive segregation system, and a number of genes are specifically required for segregating nonexchange chromosomes [2]. In this system, paired blocks of homologous heterochromatin take the place of chiasmata to maintain chromosomal associations until prometaphase [3].

All recombination in *Drosophila* is completed during prophase in the first ∼36 hours of oogenesis. After the synaptonemal complex disassembles at the end of pachytene, the chromosomes remain in prophase for approximately 3–4 days. Finally, prometaphase begins in late oogenesis with germinal vesicle breakdown (GVBD) followed by chromatin-mediated spindle assembly [1]. Mature oocytes then arrest at metaphase I with nonexchange chromosomes balanced separately on opposite sides of the spindle [4]. The meiotic spindle then remains arrested at metaphase I until the oocyte passages through the oviduct, a process that can take from 2 hours to several days [4]. However, live imaging reveals that in early prometaphase the movement of achiasmate chromosomes is highly dynamic. The two homologs move back and forth along the same arc of the spindle, such that both homologs, while still physically associated, are often positioned on the same half spindle [4,5]. This presence of physically associated *X* chromosomes on the same side of the spindle during prometaphase was first observed in fixed images from *nod* oocytes [4]. More recently, it was also documented in *ald* oocytes [5] and in *FM7/X* wild-type oocytes. This back-and-forth movement of achiasmate bivalents is usually completed within the first several hours after GVBD in these oocytes, after which the nonexchange chromosomes achieve a stable balance on opposite sides of the spindle (S. E. Hughes, J. L. Cottita, and R. S. Hawley, unpublished data).

**Figure 1:**
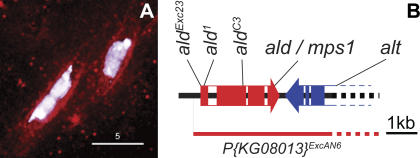
*ald* Mutants and Their Effects (A) An oocyte nucleus from a *yw; ald^C3^/ald^Exc23^; pol* female, showing DNA (white) and tubulin (red). There is complete progression past metaphase I arrest, the chromosomes have separated into two masses, and each mass possesses its own individualized tubulin spindle. (B) A map of the *ald* locus showing the location of the mutants used in this paper. *ald^Exc23^:* An incomplete excision of *P{GS:13084}* that leaves 130 nt from the 5′ end of the P element, inserted after the 14th nt of the 5′ UTR. This allele has good viability and fertility but high levels of NDJ and very low protein expression (see Figure 5B). *ald^1^:* A single nucleotide change causing a hypomorphic missense mutation (R7H). *ald^C3^:* A 9-codon deletion (Δ369–377) near the kinase active site, resulting in a semi-lethal allele similar to other *ald* null alleles. *P{KG08013}^ExcAN6^* (also referred to as *Df(3R)AN6*): An imprecise excision of a P element that was inserted in the large intron of the next downstream gene to *ald*, *CG18212*/*alt* [44]. This excision retains 3782 nt of the 5′ end of the P sequence, then deletes approximately 8 kb of sequence (red bar), from the rest of the 3′ end of the P element to 165 nt upstream of the *ald* transcription start site; 98 nt of exon 2 of *ald* is present, reversed, at the excision site. This excision deletes all of the *ald* and *alt* protein coding sequences; *alt* mutants alone were previously shown to have no detectable effect on meiosis [5].

In order to understand the mechanism by which meiotic progression is delayed sufficiently to allow the completion of this process, and thus properly partition the achiasmate homologs on opposite half spindles, we have been studying the Drosophila ald gene. Hypomorphic mutations in the *ald* gene cause high levels of achiasmate nondisjunction (NDJ) [6] and, based on the analysis of fixed images, appear to allow the precocious entry of oocytes into anaphase I [5]. *ald* was first identified in a screen for meiotic mutants causing NDJ [6]. Although the primary defect observed in *ald* oocytes was in the segregation of nonexchange chromosomes, this mutation had the unusual property of causing exchange chromosomes to nondisjoin at a low rate, as well. Cytological studies of *ald* mutant oocytes revealed a high frequency of chromosomes that appeared to have precociously entered anaphase I (Figure 1), and in which achiasmate homologs were often seen as still physically associated and thus perhaps “trapped” on one side of the spindle [5].

An explanation for these curious genetic and cytogenetic findings presented itself when *ald^1^* was shown to be a missense mutation in the *Drosophila* homolog of *mps1* [5]. Mps1 is a widely conserved kinase present in most model organisms (except nematodes) and is a key component of the meiotic and mitotic spindle assembly checkpoint (SAC) [7]. This checkpoint ensures proper alignment of kinetochores on the developing spindle, through monitoring of microtubule attachment, kinetochore tension, or both [8], and includes other conserved kinetochore-associated proteins including Bub1, BubR1, Bub3, and Mad2 [9]. Improper alignment results in the inhibition of the anaphase promoting complex, and ultimately the retention of sister chromatid cohesion. Without Mps1 kinase activity, the SAC cannot be activated in the presence of misaligned chromosomes. Therefore, the anaphase promoting complex degrades Securin, which frees Separase to cleave Cohesin, and nuclei spontaneously bypass normal arrest once sister chromatid cohesion is released [9].

Based on these observations, one could propose that the meiotic misbehavior observed in fixed images of *ald* oocytes is explained by the entry of these oocytes into anaphase prior to the time required to properly balance achiasmate homologs on opposite sides of the spindle. We show below that this is indeed the case. Visualization of meiosis in *ald* oocytes by live video microscopy revealed that shortly after spindle formation, the DNA failed to remain as a single mass at the metaphase plate, and soon entered an anaphase-like configuration where first the chromosomes and then the spindle appeared to divide into two masses, similar to what is seen in fixed images. This entry into anaphase took less time than achiasmate homologs often require to achieve proper orientation, and was never observed during prometaphase in wild-type oocytes.

The proper assembly of kinetochores is required for many cellular processes, including the SAC and chromosome attachment to microtubules [10]. Kinetochore structure has been extensively studied in mitosis, and while it can be roughly broken down into an inner and outer kinetochore plate, this likely is oversimplifying the complexity of a structure that incorporates the ∼65–70 component proteins identified to date in yeast [10]. While orthologs for all these proteins have not yet been identified in metazoans, the larger kinetochores of these organisms likely contain an equal or greater number of components. The inner centromere includes proteins such as Aurora-B and Incenp [11], which are chromosomal passenger complex proteins. Aurora-B and Incenp are required for the incorporation of outer kinetochore proteins, such as those involved in the SAC, and during cell division migrate to the central spindle [12]. Mps1 protein has been shown to exhibit mitotic kinetochore localization in *Xenopus*, mice, yeast, humans, and flies [9,13–16]. The kinetochore localization is dynamic, as fluorescence recovery after photobleaching shows the protein cycles rapidly through kinetochores with a half-life of ∼10 s in yeast [17]. We show here that Ald also localizes to the outer kinetochore in meiosis I in female *Drosophila*. Curiously, these outer kinetochore structures are sometimes, but not always, highly distended, and in such cases are well-separated from the innermost centromere protein Cid, the centromere-specific histone H3 variant [18].

In addition to its localization to the outer kinetochore, we also observed cytoplasmic filaments containing Ald, which appear to be forming at the same time as GVBD. While these filaments are not observed in mitotic cells, similar filaments were observed by GFP fluorescence in live oocytes bearing a *polo-GFP* transgene. Polo is a protein kinase that is a central regulator of many aspects of cell cycle progression through mitosis and meiosis, including regulation of Cdc25, Scc1, and the anaphase promoting complex (reviewed in [19–22]). Fixed oocytes demonstrate colocalization of Polo-GFP and Ald, confirming that these proteins are present in the same filaments. Examination of Polo-GFP localization in flies also mutant for *ald* demonstrates that Polo-GFP localization to filaments is largely unaffected, indicating that the filaments do not appear to require Ald to form. However, localization of known structural proteins, including actin, tubulin, Anillin, four septins, and two lamins failed to identify any structural protein underlying these filaments. As Ald and Polo are both kinetochore components, localization of other kinetochore-associated proteins found that BubR1 also localized to filaments in a minority of cases. However, localization of the other kinetochore components Bub1, Bub3, Mad2, Incenp, and Aurora-B did not find these proteins associated with these structures.

## Results

### Live Imaging of Meiosis in *ald* Mutant Oocytes Reveals Early Release into Anaphase

**Figure 2:**
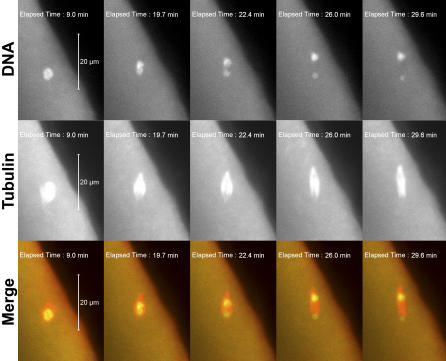
*ald* Mutant Oocytes Fail to Arrest at Metaphase I Frames from live imaging of an *FM7/yw; ald^1^/Df(3R)AN6* oocyte nucleus entering anaphase shortly after spindle formation, with no metaphase arrest. Time is relative to completion of GVBD. The spindle is tilted relative to the plane of focus, so the lower half of the spindle is extending below the bottom of the z-stack. Note that the DNA initially is a single mass, and the nonexchange *X* and *4* chromosomes have not yet moved out from the main chromosome mass. Top row: DNA (Oli-Green dye). Middle row: rhodamine-tubulin (red). Bottom row: merge.

Live imaging of meiosis in *ald* mutant females confirmed that the loss of the SAC results in premature entry into anaphase. As in wild type, GVBD occurs, and the spindle begins to form around the oocyte nucleus. However, while wild type then proceeds to elongate the spindle while maintaining the chiasmate chromosomes at the metaphase plate, oocytes from *ald* mutant females do not appear to reach a stable metaphase arrest. Instead, as exemplified by individual movie frames in Figure 2 (see Video S1 for the full movie), shortly after the spindle forms the chromatin begins to separate into separate masses, as if it were prematurely losing sister chromatid cohesion. This division is not always complete, and can appear aberrant. In some oocytes, the chromatin mass elongates and appears to partially split, but remains connected by thin threads of DNA, or large DNA masses can even move back and forth across the spindle (unpublished data). Frames from these movies resemble figures obtained by studying fixed oocytes from *ald* mutant females [5], suggesting that these results are not artifacts of either fixation or live imaging. This bypass of arrest was not observed in live oocytes bearing *nod* or *mtrm* mutants (unpublished data), indicating that the defect of maintaining metaphase I arrest is not a general feature of mutations that affect nonexchange chromosomes.

This ready progression of *ald* mutant oocytes into an anaphase-like state is not seen in *ald^+^* wild-type females, regardless of whether or not the *X* chromosomes are achiasmate (S. E. Hughes, J. L. Cottita, and R. S. Hawley, unpublished data). Out of six *FM7/X* achiasmate oocytes monitored for at least 45 min, plus an additional four *FM7/X* oocytes monitored for at least 1 h, we never observed a wild-type oocyte bypassing metaphase arrest. This is despite the fact that monitoring of some oocytes began with fully formed spindles. These movies therefore underestimate, potentially by several hours, the time expired since GVBD. Additionally, the longest an *FM7/X* oocyte was observed after imaging began was over 4 h, and it was able to maintain arrest for that duration. Therefore, out of ten wild-type oocytes monitored for at least the time approximately required to properly partition achiasmate chromosomes, none were observed to bypass metaphase arrest. Furthermore, of an additional 40 *FM7/X* and *X/X* wild-type oocytes imaged for shorter durations in various stages of prometaphase, none were observed to have bypassed metaphase arrest.

As noted above, pairs of nonexchange chromosomes can temporarily reside on the same half of the spindle prior to achieving proper arrest on opposite sides of the spindle. If entry into anaphase occurred before the achiasmate homologs were properly positioned on opposite half spindles, as is suggested by the fixed images in Gilliland et al. [5], then both chromosomes would be “trapped” on the same side of the spindle, and would both go to the same pole, resulting in NDJ. This configuration was observed in fixed images, where both autosomes completely separated from their homologs, and the two *X* chromosomes were both present on one side of the spindle (Figure S1). Therefore, the sensitivity of nonexchange chromosomes to the loss of the SAC can be explained by these chromosomes being forced to segregate before proper biorientation can be achieved.

Different *ald* genotypes can have very different rates of achiasmate NDJ, ranging from a few percent up to completely random segregation [5,6, and our unpublished observations]. The mechanism underlying this range of phenotypes could be due to the more severe genotypes losing sister chromatid cohesion earlier, which would trap more nonexchange chromosomes before completing biorientation. Alternatively, more severe alleles could be more likely to lose cohesion, without affecting the duration of prometaphase. To distinguish between these two possibilities, we imaged meiosis in *FM7/X* oocytes from three different *ald* genotypes. A total of 35 oocytes were imaged from these three genotypes, of which 12 were observed exiting GVBD, and could therefore be used to determine the duration before chromosome separation could be seen. Those oocytes that did not include GVBD could still be assayed to determine if they underwent premature sister chromatid separation. The two strongest mutant genotypes, *ald^Exc23^/Df(3R)AN6* and *ald^1^/Df(3R)AN6,* were observed to undergo premature separation in all oocytes, while the weakest genotype, *ald^1^/ald^1^,* underwent separation in nine of 21 oocytes (Table 1). However, the duration from the end of GVBD to the first evidence of chromosome separation in these genotypes did not correlate with the severity of the genetic defect, as the genotypes with the strongest and weakest genetic effects both had similar durations that were more than twice as long as the intermediate genotype. Therefore, it appears that the rate of meiotic errors in *ald* mutant females is linked to an increased likelihood of premature release of sister chromatid cohesion, rather than just a shortening of prometaphase duration.

**Table 1:**
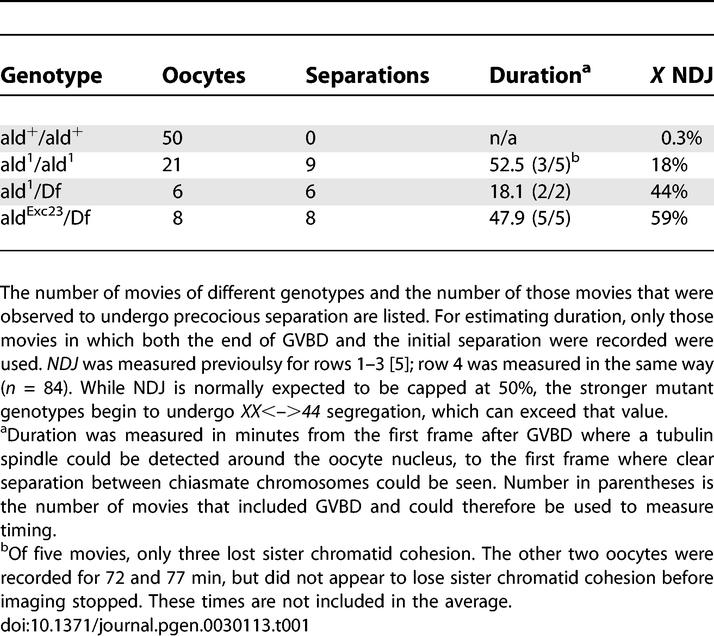
Imaging of Different *ald* Genotypes

### Ald Localizes to Outer Kinetochores in Mitosis and Meiosis

**Figure 3:**
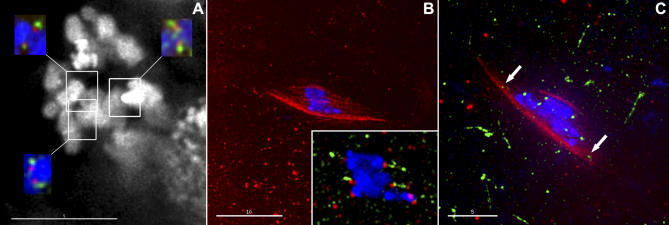
Cid and Ald Localize at *Drosophila* Kinetochores in Mitosis and Meiosis This colocalization shows the spatial relationship of the inner and outer kinetochore proteins, with Cid marking the inner kinetochore and Ald marking the outer. (A) A maximum intensity stack projection of DAPI staining in two adjacent colchicine-treated *w^1118^* larval neuroblast cells arrested at metaphase. Due to overlapping chromosomes, we have presented single optical sections of the stack (insets). Each inset is from a different optical section, with the connected border showing the location in the overall image. Each inset shows localization in a pair of sister chromatids of DNA (blue), Cid (red), and Ald (green) localization. In each case, the inner kinetochore protein Cid localized closer to the DNA than the outer kinetochore protein Ald. (B) A prometaphase oocyte nucleus from *FM7/yw*, showing DNA (blue) and the tubulin spindle (red). Inset: a 2× enlargement of the same nucleus, showing DNA (blue), Cid (red), and Ald (green). Note the extended Ald staining at the kinetochores. (C) A prometaphase oocyte nucleus from *FM7/yw,* showing DNA (blue), tubulin (red), and Ald (green). Note that the kinetochores are round foci, although the spots for the four chromosomes (arrows) are well-separated from the DAPI staining for those chromosomes.

To further characterize the role of Ald in female meiosis, we developed affinity-purified polyclonal antibodies against this protein. Previous work on *mps1* from other species as well as *Drosophila* demonstrated kinetochore localization during mitosis. By labeling with DAPI and antibodies against Ald and Cid, the *Drosophila* centromeric histone H3 variant [18], we were able to confirm that Ald localizes to the outer kinetochore in mitotic (Figure 3A) and meiotic (Figure 3B) nuclei. The Ald staining was clearly distinct from that of Cid, and was usually well out from the region of DAPI staining.

While the Ald staining sometimes appeared as round foci at the outer tips of the chromosomes during prometaphase (Figure 3C), it was also observed to be quite distended, with multiple foci extending in a line from 0.18–0.52 μm away from the Cid focus in mitosis, and 0.81–1.65 μm in meiosis. No DAPI fluorescence is detectable in this distended Ald staining, but we cannot rule out the possibility that DNA is present and undetectable (perhaps by insufficient DNA being present to detect by fluorescence relative to the nearby brightly stained main chromosomal mass, or if the extended DNA is in a configuration with which DAPI molecules cannot efficiently intercalate [23]).

### Ald Localizes to Long Filaments during Female Meiosis

**Figure 4:**
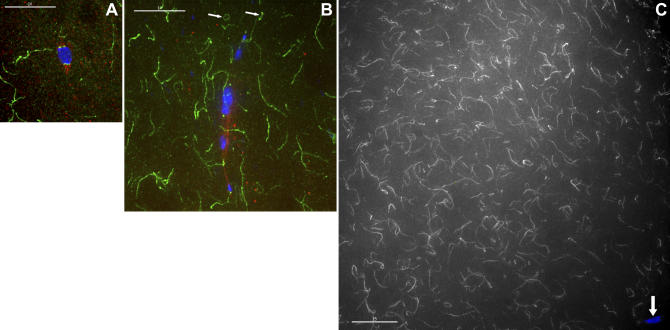
Ald Forms Extended Filaments in Meiotic Figures (A) An early prometaphase oocyte, showing kinetochore localization at the two ends of the nascent meiotic spindle, as well as nearby filaments. DNA (blue), Ald (green), and tubulin (red). (B) A *yw; pol* oocyte that has achieved metaphase arrest; the *X* chromosomes in this figure were spontaneously achiasmate, which happens 6%–9% of the time. Arrows: cytoplasmic filaments that have formed complete rings (which can be clearly seen in Video S2). (C) A 60× image of a *FM7/yw; pol* oocyte, showing Ald filaments are present throughout the oocyte. For clarity, the Ald immunofluorescent signal is shown as grayscale, while the oocyte nucleus is still shown in blue (arrow). The oocyte nucleus is in the lower right corner, showing that this oocyte is clearly in prometaphase.

**Figure 5:**
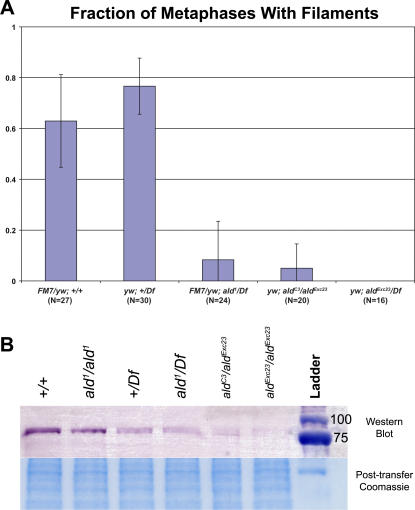
Quantification of Filament Abundances and Protein Levels (A) Frequencies of filament abundances. Meiotic spindle figures from fixed oocytes stained with DAPI, anti-tubulin, and anti-Ald antibodies were collected from the indicated genotypes, and scored on whether at least one Ald filament was visible in the optical stack. To ensure oocytes had completed GVBD and to control for antibody penetration, only figures with tubulin staining were scored. The total number of images for each genotype is indicated on the axis. It is not known why some wild-type figures had filaments while others did not, as all figures for each genotype were from the same ovary prep. In addition to fewer images having filaments, the abundance and length of the filaments in the genotypes lacking at least one wild-type copy was also reduced relative to wild type (unpublished data). For descriptions of mutant genotypes, see Figure 1B. Error bars: 95% confidence intervals. (B) Western blot denatured total protein extract from 1–5-h-old eggs laid by fertilized females of the indicated genotypes (5 eggs/lane), with the post-transfer gel stained with Coomassie as loading control. Ald protein (predicted size ∼76 kDa) was visualized using alkaline-phosphatase conjugated secondary antibody. The wild-type and *ald^1^* homozygotes had similar amounts of signal, as did the wild-type and *ald^1^* hemizygotes. The other mutant genotypes (described in Figure 1B) had greatly reduced levels of protein. Complete null alleles could not be used due to the need for females healthy enough to lay eggs.

**Figure 6:**
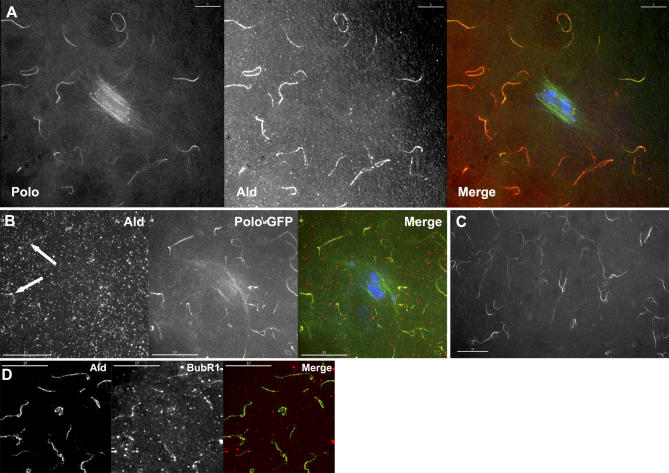
Polo and BubR1 Are Also Present in Ald-Containing Filaments (A) Polo-GFP/Ald colocalization A fixed oocyte from a *polo-GFP* homozygous female, showing that only Polo localizes along the meiotic tubulin spindle, while both Ald and Polo colocalize to kinetochores and along the cytoplasmic filaments. The chiasmate chromosomes appear slightly abnormal, possibly due to overexpression of Polo, but achiasmate *X* NDJ was no more than 1%–3% (our unpublished observations). (B) Polo-GFP still highlights filaments in *ald* mutant oocytes. A fixed oocyte from a *polo-GFP/+; ald^Exc23^* female. This *ald* allele greatly reduces, but does not eliminate, the dosage of wild-type Ald present (see Figure 5B). Ald staining is mostly in discrete foci, some of which are close enough together to be interpreted as filaments (arrows). However these filaments still contain Polo-GFP fluorescence, which appears undisturbed by the reduction in Ald dosage. (C) Unfixed Polo-GFP also highlights filaments GFP fluorescence from an unfixed *polo-GFP* oocyte, with filaments qualitatively similar to those seen in fixed images. Due to the lack of a DNA marker, the location of these filaments relative to the oocyte nucleus could not be determined, but based on Polo-GFP localization to the spindle (see Figure 6A), the oocyte nucleus does not appear to be in the imaged region. (D) BubR1 can also highlight filaments. In ten out of 33 *FM7/X* oocytes, BubR1 was found to weakly highlight some filaments visualized by Ald antibody. This figure represents the best staining observed; in most oocytes the staining was weaker (but still detectable) and only present in a subset of filaments.

In addition to the kinetochore localization of Ald protein, the antibody also highlighted numerous cytoplasmic filaments in prometaphase oocytes (Figure 4). The filaments were found throughout the cytoplasm, and did not appear to be associated with any membranes or vesicles. That these filaments were due to Ald localization, and not cross-reaction of the antibody to other proteins, could be seen by the reduction in frequency that filaments were observed in several different mutant backgrounds (Figure 5A). Serendipitously, work in the lab with a *polo-GFP* transgene [24] also revealed similar filaments of GFP fluorescence in prometaphase oocytes; staining of *polo-GFP* oocytes with the anti-Ald antibody demonstrated that Polo-GFP and Ald were found in the same structures (Figure 6A). To determine if these filaments require Ald protein, we fixed and stained oocytes from *polo-GFP/+; ald^Exc23^* females. This *ald* allele produces greatly reduced levels of Ald, but still has a detectable amount of wild-type protein (Figure 5B). In these oocytes, the outline of the filaments was barely detectable by Ald staining in a few oocytes, but appearance of the filaments visualized by Polo-GFP appeared to be unaffected (Figure 6B). This indicates that the filaments do not require Ald to form, and may contain an underlying structural protein. However, the filaments did not costain with antibodies against the structural filament proteins tubulin (Figure 4); actin; the contractile ring protein Anillin; the septins Sep1, Sep2, Sep4, or Peanut; or the nuclear membrane proteins Lamin or Lamin-C (Figure S2).

Several lines of evidence indicate that these filaments are not artifacts of fixation or of the GFP construct. First, very similar GFP filaments could be observed in live, unfixed oocytes dissected from Polo-GFP females (Figure 6C). Second, Ald staining filaments were first observed in flies that did not carry Polo-GFP. Finally, a careful examination of figures from a previous study using an anti-Polo antibody in oocytes also reveals filaments of Polo staining [25 and K. McKim, personal communication]. While we have not tested the Ald and Polo antibodies in the same prep, the fact that Polo forms filaments with and without the GFP fusion construct makes it likely to not be artifactual.

**Figure 7:**
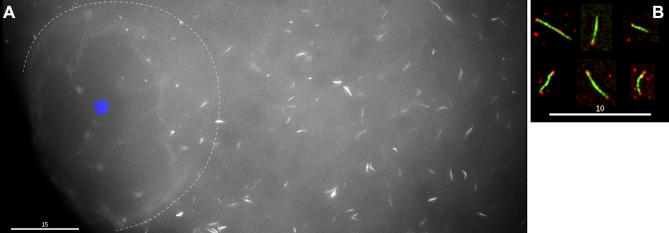
Polo-GFP Filaments Appear to Form at GVBD (A) A 60× image of a *polo-GFP* oocyte, apparently fixed in early GVBD. Note the germinal vesicle is still present (dotted outline), but with “ruffling” of the membrane, indicating the germinal vesicle is beginning to break down. Only GFP (grayscale) and DAPI (blue) are shown for clarity; Ald (Cy3) showed colocalization to filaments, and tubulin (Cy5) showed no detectable spindle formation around the oocyte nucleus. Short and relatively uniform Polo-GFP filaments are present throughout the cytoplasm, but not within the germinal vesicle. Those filaments within the dotted outline were in different focal planes than the membrane, and were clearly outside the germinal vesicle. When compared to most oocytes with filaments (compare to wild type in Figure 4C, and Polo-GFP in Figure 6B), the cytoplasmic filaments are much shorter (most well under 5 μm). (B) Individual filaments from a *FM7/yw* oocyte, apparently fixed during GVBD, stained with anti-Ald (green) and anti-BubR1 (red) antibodies. Note the very similar lengths of the filaments and the presence of BubR1 foci at the tips of the Ald filaments.

It also appears that these filaments are polymerizing throughout the oocyte cytoplasm concurrently with GVBD. While we have never observed filaments in oocytes that were clearly pre-GVBD, to date we have identified three oocytes that appear to have been fixed in the process of breaking down the germinal vesicle, based on the shape of the karyosome and the “ruffled” appearance of the membrane. In these oocytes (one carrying Polo-GFP and two wild type), the filaments are shorter and more uniform in length than usual (Figure 7A). This uniformity suggests that filament formation is triggered by a diffusible signal that is able to transverse the cytoplasm rapidly relative to the rate of filament formation. Furthermore, in these oocytes the filament formation has not extended into the area of the germinal vesicle. We also note that the one wild-type oocyte we have at this stage that was stained with anti-BubR1 antibodies has several notable features in the BubR1 staining. First, while most of the BubR1 staining is found in punctate foci, inside the germinal vesicle the BubR1 staining is still diffuse. This suggests that the same set of changes that result in the Ald/Polo filament formation also moves the BubR1 protein into discrete foci. Furthermore, many (but not all) Ald filaments are associated with BubR1 foci at one or both ends (Figure 7B).

These filaments may be similar, at least in some respects, to filaments that were previously observed in C. elegans oocytes [26]. In that study, the proteins KNL-1, KNL-3, MIS-12, NDC-80, Nuf2^HIM-10^, and BUB-1 were reported to localize to short filaments that appeared only in female meiosis I, and disappeared by meiosis II. We note that there are several qualitative differences between those filaments and the ones highlighted by our antibody, most significantly that the worm filaments are associated with the egg cortex and spindle while the fly filaments were found throughout the oocyte cytoplasm and did not appear to associate with the spindle at all. To determine if the fly filaments were also dispersing by meiosis II, we examined live zygotes from Polo-GFP mothers at 9–15 min post-oviposition (mean time 11.9 min). This is after oocytes have been fertilized and activated, and will be undergoing meiosis II [27]. We found no cases (0/23 zygotes) where GFP filaments could be observed, which is consistent with the filaments disassembling by meiosis II. As a control, after egg collection was finished, egg-laying females were dissected and their oocytes visualized, which confirmed that filaments could be seen in pre-activation oocytes.

**Figure 8:**
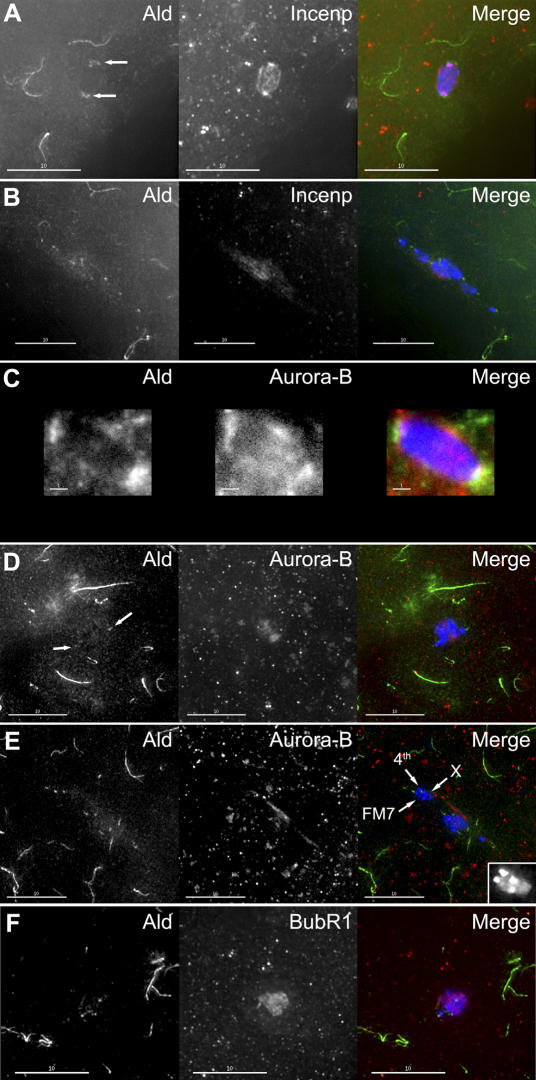
Incenp and Aurora-B Colocalize to the Meiotic Spindle but Not to Ald Filaments All oocytes are from *FM7/yw; pol* females, hybridized with anti-Ald plus either anti-Aurora-B, anti-Incenp, or anti-BubR1 antibodies. Each oocyte shows Ald, (left panel); Aurora-B, Incenp, or BubR1 (middle panel); and DAPI (right panel). The high background off of the oocyte nucleus is from the large amount of maternally loaded protein. In no figure were Aurora-B or Incenp protein found to localize to the Ald cytoplasmic filaments. (A) An early prometaphase oocyte nucleus, showing inner-kinetochore Incenp localization and less-punctate staining along the spindle. Arrows: kinetochore-associated Ald foci. (B) A metaphase-arrested oocyte nucleus, showing no Incenp kinetochore localization and diffuse localization near the metaphase plate. (C) A magnification of the chromosomes in an early prometaphase oocyte nucleus, showing localization of Aurora-B to the kinetochores and spindle. While this staining was weaker than Incenp, and not greatly above background, in the full z-stack the brightest kinetochore localization was clearly in the same optical section as both the tips of the DNA and the Ald kinetochore foci. (D) A mid-prometaphase oocyte nucleus showing Aurora-B localization along the metaphase plate, but not to the cytoplasmic Ald filaments. Arrows: kinetochore-associated Ald foci. (E) A mid-late prometaphase oocyte nucleus, with a stripe of Aurora-B localization to one side of the spindle midbody. Note that both *X* chromosomes are physically associated with each other and one Chromosome *4* on the same side of the spindle (labels). *X* and *FM7* can be discriminated by the pattern of DAPI staining of heterochromatin. Inset: A 2× enlargement of the DAPI signal for the two *X* chromosomes and one *4* chromosome. (F) A mid-prometaphase oocyte nucleus with Ald and BubR1 staining. The spindle is pointing into the plane of the image. Note the Ald at kinetochores and the localization of BubR1 on the chromosomes.

Identifying proteins in common to the filaments in both species is difficult, as there is no *mps1* ortholog in C. elegans, and to the best of our knowledge Polo has not been localized in oocytes in that species. Similarly, there are no fly orthologs for KNL-1, KNL-3, MIS-12, NDC-80, or Nuf2^HIM-10^ (http://www.flybase.org). The only proteins that Monen et al. [26] localized in *C. elegans,* which also have orthologs in *D. melanogaster,* were Aurora-B and Bub1. We were able to stain oocytes with antibodies against Aurora-B and Bub1, as well as the kinetochore-associated proteins Incenp, Bub3, Mad2, and BubR1 (Figure 8). Of these, only BubR1 localized to the filaments, and then weakly and only in about 30% of oocytes (Figure 6D). However, this may represent either a case of paralogy or a transfer of functionality between these genes, as the two Bub1-like genes in flies are more closely related to each other than to their orthologs in other species [28].

While these proteins clearly did not colocalize to the Ald-containing filaments, some of them did show localization to the meiotic spindle (Figure 8). Interestingly, the localization of both Aurora-B and Incenp proteins changed from early prometaphase to metaphase arrest. In early prometaphase, Incenp was found at kinetochores (closer to the DNA than Ald) as well as patchily distributed throughout the spindle (Figure 8A). By metaphase arrest, the kinetochore association is lost, and the protein is found broadly distributed along the middle of the spindle (Figure 8B). Similarly, Aurora-B associated weakly with kinetochores and the outer edge of the spindle in early prometaphase (Figure 8C), then along the metaphase plate in mid-prometaphase (Figure 8D). By metaphase arrest Aurora-B was found in a relatively narrow strip along only one outer edge of the spindle (Figure 8E). For both proteins, Ald kinetochore localization persisted after Aurora-B/Incenp was no longer found at kinetochores. This finding of early kinetochore localization of Aurora-B and Incenp is consistent with the finding that these two proteins are required for recruiting many kinetochore components, including Mps1, during mitosis [12]. In our hands, the Bub1, Bub3, and Mad2 antibodies did not localize to the meiotic spindle or the prometaphase kinetochore. That this was not simply antibody failure could be seen by localization to other structures, such as Bub1 staining present inside the germinal vesicle before breakdown, or Mad2 localizing to follicle cell nuclei surrounding the oocyte (unpublished data). BubR1 did localize to kinetochores in 21% (6/29) of oocytes, but also localized to the surface of the chromosomes (Figure 8F).

## Discussion

### The Role of the SAC in Facilitating the Proper Co-orientation of Achiasmate Bivalents

The progression of meiosis is a tightly temporally regulated event [29] and the study of cell cycle regulation is greatly enhanced by being able to visualize meiosis in living cells. In our study, a defect in a kinetochore component results in the precocious entry into anaphase, presumably as a result of the early activation of Separase, as well as the mis-segregation of nonexchange chromosomes. We explain the greater sensitivity of nonexchange chromosomes to *ald* mutants by noting that while chiasmate chromosomes appear to co-orient immediately upon GVBD, nonexchange chromosomes require more time to achieve proper biorientation. The test of this hypothesis lies in the relative timing of these two events, data which are straightforwardly obtained from live imaging but difficult, and considerably more ambiguous, to obtain only from the examination of fixed images.

As we have observed, *ald* mutant oocytes can enter an anaphase-like configuration practically as soon as the spindle is formed, so we believe that this requirement is satisfied. This interpretation is strengthened by the three genotypes we examined by live imaging. For the two genotypes with the highest rates of NDJ *(ald^1^/Df(3R)AN6* and *ald^Exc23^/Df(3R)AN6),* every oocyte examined appeared to undergo premature separation, while the weakest genotype (*ald^1^/ald^1^)* only lost cohesion in 43% of oocytes. This is consistent with the NDJ data, as homologs segregating at random are expected to nondisjoin half of the time, and so if the failure to maintain sister chromatid cohesion is the cause of the NDJ, it should occur at approximately twice the rate of NDJ. While the duration of prometaphase did not correlate with the propensity to nondisjoin, this may reflect a difference between the alleles. The *ald^1^* oocytes contain only hypomorphic protein, while the *ald^Exc23^* oocytes still contain a small amount of wild-type protein (Figure 5).

We furthermore note that strong *ald* alleles also induce chiasmate NDJ at high levels, a fact underscored by the mutant screen that recovered *ald^C3^* (S. L. Page and R. S. Hawley, unpublished data). This germline clone screen only recovered mutants that could cause a chiasmate autosome to nondisjoin, and *ald* was the most commonly hit gene, with four out of 12 recovered mutants containing new alleles of *ald*. The mechanism cannot be the same as that for nonexchange chromosomes, as chiasmate chromosomes appear to be properly co-oriented after GVBD, without the back-and-forth movement observed for *FM7/X*. One possibility is that the loss of sister chromatid cohesion when the SAC is impaired causes all chiasmata to resolve during early prometaphase. This would result in a situation analogous to mutants such as *meiW68,* the *Drosophila* homolog of *spo11,* where recombination has been completely abolished, which also cause high levels of NDJ [30]. Consistent with this interpretation, metaphase I arrest is also bypassed in oocytes with mutants that abolish crossing over [31] as well as chromosomal configurations where chiasmata form but do not establish bipolar tension [32].

### Ald and the Outer Kinetochore

We have also extended the previously known mitotic kinetochore association of the Ald protein to female meiosis. It was surprising that in some, but not all, cases, the kinetochore staining of Ald clearly extended well outside the centromere region identified by Cid. One possibility is that the very rapid cycling of Ald through the kinetochore [17] proceeds by transport of the protein along attached microtubules. If this is the case, the extended kinetochores highlighted by Ald staining represent protein that has been released from the kinetochore but has not yet detached from the microtubule. Alternatively, the finding of such kinetochores evokes the model of stretched centromeric DNA observed during mitosis using the LacO/LacI-GFP system in budding yeast [33–36]. For example, He et al. found that LacO arrays inserted closer than 9 kb away from the centromere were found to transiently separate from their homolog by up to 0.8 μm during metaphase, while arrays inserted 13 kb or further away from the centromere were separated by only 0.3 μm [33]. They interpreted this finding as indicating the centromere-proximal chromatin was being stretched, possibly as part of how the kinetochore senses tension. In light of this finding, an alternative interpretation of our extended Ald staining result is that tension on a kinetochore is able to change its shape, and therefore the cytology may reflect the SAC being in an on or off configuration. As checkpoint signaling requires the kinase activity of Mps1 [9], finding this protein along the extended kinetochore structure means it is well positioned to perform this role.

However, we must note that we observed neither DAPI fluorescence nor Cid localization to these extended kinetochores, and cannot prove that there is DNA present in these structures. It may be that the structure highlighted by extended Ald staining was composed solely of proteins, and represents a kinetochore that is structurally different from the mitotic kinetochore. The *Drosophila* female meiotic kinetochore has not previously been examined by electron microscopy, and given the lack of centrosomes, the female meiotic kinetochore may have important structural differences as well. If this is the case, then the *Drosophila* ovary should prove well suited to the study of this kinetochore. In addition to the possibility of monitoring the relative positioning of different proteins on the spindle via immunofluorescence, the well-established use of germline clones would allow for studying the effects on meiosis of mutations in genes that would be lethal during development [37].

We also note that our localization of Aurora-B and Incenp is different from what was previously reported in mitosis. In mitosis, Aurora-B is a chromosomal passenger protein that is essential to the function of the SAC, and migrates from the kinetochore to the spindle midzone at the metaphase/anaphase transition [38]. Here, we show that in female meiosis, this migration happens early in prometaphase, prior to checkpoint inactivation, and localizes to either the midzone (for Incenp) or to a stripe along only one side of the spindle (for Aurora-B). That these two proteins form a different final configuration shows they are no longer present in the same complex at metaphase arrest. We also note that our localization is slightly different from that previously reported by Jang et al. in *Drosophila* oocytes [25]. In that study, they were unable to find localization of Aurora-B and Incenp to the kinetochore during prometaphase, and they did not see a stripe of Aurora-B staining. We found kinetochore localization only during the very earliest stages of prometaphase, before the *4* chromosomes have begun moving out from the main chromosomal mass, which was an earlier stage than the images presented in their paper. Also, our study used achiasmate *FM7/X* oocytes, while Jang et al. used chiasmate *X/X* oocytes. That the Aurora-B stripe at metaphase arrest appeared along the side of the oocyte that the nonexchange *X* chromosomes were associated with suggests that this is a feature of achiasmate chromosomes, and may be why Jang et al. did not observe it. It also suggests that Aurora-B may play a role in balancing nonexchange homologs on the meiotic spindle.

### Filaments of Outer Kinetochore Kinases in the *Drosophila* Oocyte

Finally, the identification of Ald-containing filaments represents a novel structure in the *Drosophila* oocyte, as well as a novel functionality for both Ald and Polo kinases. That these filaments could be found in fixed and unfixed *polo-GFP* oocytes, as well as fixed wild-type oocytes, indicates that they are neither an artifact of fixation nor of the GFP construct. While both Polo and Ald are kinetochore-associated proteins, of the other kinetochore-associated proteins we examined (Cid, Aurora-B, Incenp, Bub1, BubR1, Bub3, and Mad2), only BubR1 localized to a minority of filaments.

The filaments appear to be sequestering Ald protein, but do not require Ald for their formation. This is clearly indicated by the *Polo-GFP/+; ald^Exc23^* oocytes, in which filaments were barely detectable by Ald staining, but appeared unaffected when visualized by Polo-GFP staining. Therefore, we conclude that the reduction of filaments in the various mutant backgrounds must be due to a failure to detect the filaments, even though the filaments are still present. The occasional filament seen in some images confirms this interpretation, as only those filaments that incorporated enough Ald protein over a small region would be classified as a linear structure. Doing the reciprocal experiment of staining for Ald in the presence of a Polo mutant cannot be done due to the sterility of those mutants, but may be possible to conduct through the use of germline clones. Interestingly, the *ald^1^* hemizygotes had fewer filaments than *ald^+^* hemizygotes despite having similar levels of protein. One possibility is that the *ald^1^* allele cannot be sequestered as efficiently as *ald^+^.* As Mps1 is phosphorylated during checkpoint activation [39], one possibility is that the *ald^1^* mutation disrupts a phosphorylation site. Indeed, there is an in silico-predicted phosphorylation site that is disrupted in the *ald^1^* mutant for the threonine-9 codon (NetPhos 2.0 score, *ald^+^*: 0.752, *ald^1^*: 0.314.) [40], which suggests that phosphorylation may be involved in regulating the incorporation of Ald into these filaments.

We are also interested in how these filaments are formed. We propose that the polymerization is triggered by a signal propagating through the oocyte, and that this signal occurs during germinal vesicle breakdown. However, what that signal is, and what the targets of that signal are, remain to be determined. It is also unclear whether the formation of filaments is necessary for the initiation of GVBD, or a downstream consequence of it. The presence of BubR1 foci at the tips of many of the filaments as they are forming also suggests that other proteins are involved in their construction; we note that BubR1 was the only protein (besides Ald and Polo) to incorporate into the filaments at all. A further question, as these filaments appear to disassemble by meiosis II, is what signal initiates the disassembly, and what proteins are required to respond to that signal. Finally, the function of these filaments is unknown. While we have no direct evidence for the function of these filaments, several possibilities suggest themselves. One possibility is that, because many SAC proteins (including Mps1 and Polo) are degraded as part of checkpoint inactivation [39], the filaments may protect the maternal load of these proteins during this stage. However, this interpretation is not consistent with the filaments dispersing by meiosis II, as the proteins would still be susceptible to degradation during the syncytial nuclear divisions. Another possibility is that the filaments allow the cell to regulate the activity of these proteins during meiosis I. One approach to answer these questions would use immunoprecipitation to isolate these filaments and determine what other proteins are present, followed by examination of mutant alleles of those component proteins. The mechanism that these filaments carry out could likely be inferred by the meiotic defects of mutants in other genes involved in filament formation.

## Materials and Methods

For live imaging of DNA and tubulin, oocytes from females aged 3–5 d in yeasted bottles with males were dissected, injected with rhodamine-tubulin (Cytoskeleton, http://www.cytoskeleton.com) and Oli-Green DNA dye (Molecular Probes, http://www.invitrogen.com), and imaged as described (S. E. Hughes, J. L. Cottita, and R. S. Hawley, unpublished data). *FM7/w; pol* females were used for wild-type oocytes, and females of the genotypes *FM7/y; ald^1^/ald^1^, FM7/yw; ald^1^/Df(3R)AN6,* and *FM7/yw; ald^Exc23^/Df(3R)AN6; pol* were used for *ald* mutants. For live imaging of Polo-GFP oocytes, females were dissected in halocarbon 700 oil on a cover slip, mature oocytes were separated from ovarioles under oil with forceps, and imaged on a Deltavision deconvolution microscope driven by SoftWorX (Applied Precision, http://www.api.com) using the FITC filter set. For live imaging of zygotes, a thin layer of grape juice agarose was cast on a microscope slide, which was then inserted into a cage containing Polo-GFP flies and no other material on which to deposit eggs. The slide was removed 5–10 min later, a drop of glycerol and a cover slip were immediately placed directly on the agarose, and any zygotes were immediately imaged.

For all fixed images, females were dissected, fixed, and antibody-hybridized as described previously [5]. Unless specifically mentioned, all images were captured with a 100× objective. Oocytes from Figure 4A and 4B were incubated with 50 nM colchicine for 10 min prior to fixation. While this treatment increased the number of metaphases that contained filaments, it was insufficient to completely disrupt microtubules, and did not change the length or number of cytoplasmic Ald filaments by more than 10% when compared to incubation-only controls, as measured using Volocity (http://www.improvision.com) image classifiers (unpublished data). Guinea pig anti-Ald primary antibody was used at 1:1,000 dilution for immunofluorescence. Other antibodies were rat anti-alpha-tubulin (Serotec, used at 1:250), mouse anti-Peanut monoclonal 4C9H4 [41] (1:4), mouse anti-Lamin monoclonal ADL195 [42] (1:1), mouse anti-Lamin C monoclonal LC28.26 [43], chicken anti-Cid [18] (1:100), and rabbit antisera generated against the following proteins: Aurora-B [11] (1:500), Incenp [11] (1:500), Anillin (1:100), Sep1 (1:50), Sep2 (1:50), Sep4 (1:100), Bub1 [28] (1:250), BubR1 [28] (preabsorbed 1:100, final 1:1,000), Bub3 (preabsorbed 1:300, final 1:9,000), and Mad2 [28] (1:100). Fluorophore-conjugated goat anti-IgG secondary antibodies were used at 1:250 dilution. Actin was visualized with Alexa-547 conjugated phalloidin used at 1:166.

For western blotting, females laid eggs on yeasted grape juice-agar plates for 4 h. Due to anesthetization, very few eggs were laid early in that time period. Up to 30 eggs were then manually transferred to an eppendorf tube, 1 μl/egg of 2× SDS-PAGE loading buffer was added, eggs were crushed with a plastic pestle, and then the tube was heated to 95 °C for 5 min and stored at −20 °C. Five microliters of these preparations were run on precast Ready-Gels (Bio-Rad, http://www.bio-rad.com), and transferred to PVDF membrane using a semi-dry transfer cell at 15 V for 90 min. After transfer, gels were preserved and stained overnight with Coomassie (Bio-Rad) as a loading control. The blot was blocked with PBSTB (PBS + 0.1% Triton X-100 + 4% dry milk powder), hybridized overnight with shaking at 4 °C in PBSTB with 1:5,000 anti-Ald antibody, washed five times for 10 min with PBSTB, hybridized as before with 1:3,000 alkaline-phosphatase conjugated secondary antibody, then visualized with BCIP + NBP.

The antigen for creating polyclonal antibodies was created by digesting the *ald* rescue construct plasmid [5] with EcoRV and BamHI and gel-purifying the 690-bp band encoding amino acids 150–379 of the protein. Bacterial expression vector pET-41a (Novagen) was digested with NotI, resected with Mung Bean Nuclease, digested with BamHI, and dephosphorylated with CIP. Ligation was done with one blunt (EcoRV + resected NotI) and one cohesive end. To place the 6×HIS tag in frame, this construct was digested with XhoI, ends resected with Mung Bean Nuclease, and religated, to induce a 4-nt deletion. The final plasmid was verified by sequencing. This construct was transformed into BL21 (DE3) cells (Invitrogen), induced with IPTG, lysed with 8 M urea, and the expressed protein was isolated with ProBOND nickel bead resin (Invitrogen). Animals were injected by Cocalico Biologicals (http://www.cocalicobiologicals.com). The same antigen protein was also used with SulfoLink columns (Pierce, http://www.piercenet.com) for affinity purification of the resultant serum per manufacturer's instructions.

## Supporting Information

Figure S1 A Fixed Image from an *FM7/X; ald^Exc23^/Df(3R)AN6* Mutant Female, Showing the Autosomes Having Partitioned into Two Groups Chromosomes are identified based on the pattern of DAPI brightness and chromosome size, assuming Chromosome *3* is larger than Chromosome *2*. Note that while the autosomes have moved into two groups, the two *X* chromosomes are both on the same side of the spindle.
(1.2 MB TIF)

Figure S2 Other Structural Proteins Do Not Colocalize with Ald Filaments All panels (except A, G, and H) are of fixed ovaries from *FM7/yw; pol* females, stained with DAPI and immunolocalized for Ald and the indicated second protein. In no case did the other protein localize to the Ald/Polo filaments. (A) A fixed oocyte from *polo-GFP* stained with Alexa-547 conjugated phalloidin to visualize actin. The oocyte nucleus is not in the imaged region. Inset: Actin staining highlighting the ring canals connecting cells in an early cyst as a positive control. (B) Anillin staining was localized primarily to vesicle-like inclusions; these showed that diffuse Ald staining was excluded (left panel, arrows 1 and 2). Note that inclusion 1 is dark in both channels, while inclusion 2 stains for Anillin. Note the Ald kinetochore spots and oocyte nucleus, just right of center. (C) Peanut was found in small loops between follicle cells around the surface of the oocyte; the Ald staining was in a different optical plane. The oocyte nucleus is not in the imaged region. (D) Sep1 staining was found in punctate foci in the cytoplasm, as well as between follicle cells (similar to Peanut, unpublished data). Note the oocyte nucleus in the lower left corner (arrow). (E) Sep2 staining was in diffuse blotches in the cytoplasm. Note the oocyte nucleus, center. Sep2 staining could also be found in a layer at the base of the follicle cells in earlier-stage oocytes as a positive control (unpublished data). (F) Sep4 staining was quite strong near the surface of the oocyte, but not within the cytoplasm. (G) An *FM7/X; ald^1^* oocyte stained with anti-Ald (red) and anti-Lamin (green) antibodies. While Lamin was not found in filaments, it did highlight nuclear membranes in the follicle cells and cysts, as expected (unpublished data). (H) An *FM7/X; ald^1^* oocyte stained with anti-Ald (red) and anti-Lamin-C (green) antibodies. While Lamin-C was not found in filaments, it highlighted nuclear membranes in the follicle cells and cysts. Additionally, starting at around Stage 6, the oocyte nucleus became increasingly highlighted by Lamin-C, with much stronger staining found by stage 10 in oocytes and retained until before GVBD (unpublished data).
(4.4 MB TIF)

Video S1 Live Microscopy Imaging of *ald* Mutants The full movie from the *FM7/X; ald^1^/Df(3R)AN6* mutant oocyte used to generate Figure 2. During the movie the spindle rotates slightly, so that the lower half of the spindle extends below the lowest optical section and disappears from view.
(2.4 MB MOV)

Video S2 Three-Dimensional Filaments around the Spindle A 3D reconstruction of the image stack used in Figure 4B, showing the three-dimensional characteristics of the Ald filaments. Note that filaments are distributed all around the meiotic spindle.
(429 KB MOV)

## Abbreviations

GVBD: germinal vesicle breakdown
NDJ: nondisjunction
SAC: spindle assembly checkpoint
